# Psychometric properties of the Knowledge and Attitudes to Mental Health Scales in a Dutch sample (KAMHS-NL): A comprehensive mental health literacy measure in adolescents

**DOI:** 10.1186/s12889-024-19371-3

**Published:** 2024-07-25

**Authors:** Janne M. Tullius, Bas Geboers, Roy E. Stewart, Yifeng Wei, Sijmen A. Reijneveld, Andrea F. de Winter

**Affiliations:** 1 Division of Community & Occupational Medicine, Department of Health Sciences, University Medical Center Groningen, University of Groningen, PO Box 196, Groningen, 9700 AD The Netherlands; 2 Department of Psychiatry, University of Alberta, Edmonton, AB Canada

**Keywords:** Avoidant coping, Help-seeking, Knowledge, Mental health, Mental health literacy, Psychometric properties, Questionnaire, Self-stigma, Stigma

## Abstract

**Background:**

Mental health literacy (MHL) is crucial for early recognition of and coping with mental health problems, and for the use and acceptance of mental health services, leading to better health outcomes, especially in adolescence. The prevalence of mental health problems among adolescents is seen as a major public health concern and MHL is an important factor in facilitating positive mental health outcomes. However, the availability of valid measurement instruments for assessing the multifaceted nature of MHL is limited, hindering the ability to make meaningful comparisons across studies. The Knowledge and Attitudes to Mental Health Scales (KAMHS) is a promising comprehensive instrument for measuring adolescents’ mental health literacy but its psychometric properties have not been explored in any other contexts than the Welsh. The aim of this study was to translate the KAMHS into Dutch, adapt it in this context, and evaluate its psychometric properties.

**Methods:**

We performed a cross-sectional study with Dutch adolescents between the ages 11–16. We translated the KAHMS and assessed its content validity using cognitive interviewing with *n* = 16 adolescents. Next, *n* = 406 adolescents were asked to fill in the translated KAMHS-NL and reference scales, on mental health (SDQ and WHO-5), resilience (BRS), and mental health help-seeking behaviors. We assessed construct validity based on a priori hypotheses regarding convergent and divergent correlations between subscales of KAMHS-NL and the reference scales. Finally, we assessed structural validity via confirmatory factor analysis and exploratory structural equation modeling.

**Results:**

The KAMHS-NL showed good content validity and satisfactory construct validity. In total, 28 of the 48 hypotheses regarding convergent and divergent correlations between the KAMHS and reference scales were confirmed. Contrary to our expectations, weak, but significant associations were found between MHL and resilience. The KAMHS showed an acceptable to good internal consistency (McDonald’s omega ranging from 0.62 to 0.84). Finally, we could generally confirm the postulated structure of the KAMHS-NL in the Dutch sample with a 5-factor solution (RMSEA = 0.033; CFI = 0.96).

**Conclusions:**

The Dutch version of the KAMHS is a valid measure for detecting differences in MHL levels in adolescents. The KAMHS is a promising instrument for assessing MHL in adolescents in a multifaceted manner in other countries which may facilitate rigorous global MHL research. The instrument therefore deserves further validation research in other settings and comparisons across various cultural contexts.

**Supplementary Information:**

The online version contains supplementary material available at 10.1186/s12889-024-19371-3.

## Background

Mental health literacy (MHL) has become a rising and evolving concept in the literature of mental health [1]. MHL refers to the competence to “understand how to obtain and maintain positive mental health; understand mental disorders and their treatments; decrease stigma related to mental disorders; and enhance help-seeking efficacy” (1, p. 2). MHL has been found to be a crucial prerequisite for early recognition, self-management of and coping with mental health problems, and the use and acceptance of mental health services [2–4]. Especially during the developmental phase of adolescence, a time period when most mental disorders have their onset, improving MHL has been identified to be an effective strategy for the promotion and prevention of mental health problems [5, 6]. For research and practice, it is important to be able to detect differences in the level of MHL among adolescents. Due to MHL being a relatively young concept, measurement tools for adolescent MHL are being developed, but are thus far not widely validated which limits the consistent measurement across studies and therefore the comparison of findings [7, 8].

Validated measurement tools of MHL capturing the full breadth of the concept are still not widely available. Existing assessment tools of MHL often lack the components of mental health resource knowledge (help-seeking efficacy) and maintenance of positive mental health essential to the more recent mental health promotion-focused definition of MHL [9–11]. Wei and colleagues revealed in a scoping review of available MHL measures that there is a strong imbalance of knowledge and help-seeking measures compared to stigma/attitude measures and, even more prominent, the overall lack of measures that measure all components of MHL at once [12]. Other systematic reviews support this finding [7, 13].

Simkiss et al. (2021)’s most recently developed MHL assessment tool “Knowledge and Attitudes to Mental Health Scales” (KAMHS) presents however a comprehensive and reliable measure for adolescents following the most recent conceptualization of MHL [14]. The KAMHS contains all components included in the most recent definition of MHL, such as help-seeking efficacy and maintenance of positive mental health, and thus differentiates itself in comparison to other tools. In addition to the commonly measured components of MHL, the KAMHS includes a subscale measuring (lack of) avoidant coping and a subscale measuring socially desirable responses (Social Desirability subscale). The subscale of (lack of) avoidant coping was included in the KAMHS by the original authors as previous research has indicated that poor coping strategies are a common barrier to help-seeking [15]. The subscale of social desirability is not a component of MHL but has been included in the KAMHS as it helps to identify individuals who are reporting an overly positive image leading to information bias. Such attributes make the KAMHS one of the most comprehensive measurement instruments of MHL for adolescents. In order to start streamlining MHL measurements and enable high quality research on adolescent MHL across contexts, such existing comprehensive instruments ought to be systematically translated, adapted and validated [7, 16].

Therefore, the current study aims to translate and culturally adapt the KAMHS, a comprehensive instrument measuring adolescents’ mental health literacy to Dutch and assess its psychometric properties.

## Methods

This study has been designed according to the COSMIN (COsensus-based Standards for the selection of health Measurement INstruments) guidelines [17]. It has been performed in two phases: (1) Translation, cultural adaptation, and content validity of KAMHS-NL, and (2) Assessment of psychometric properties of KAMHS-NL.

Both phases of this study were performed in accordance with the Helsinki Declaration. The study was deemed exempt from human subjects’ review (non-WMO study) by the Medical Ethical Committee of the University Medical Center Groningen (no. M20.252893). Informed consent was obtained at the beginning of the interview or survey from the participants. For participants younger than 16, additional (passive) informed consent was obtained from their legal guardian. Legal guardians were informed about the study via e-mail with information sheets and were given the possibility to withdraw their child from participation by responding via e-mail.

### The Knowledge and Attitudes to Mental Health Scales (KAMHS)

The KAMHS is a reliable multifaceted self-report questionnaire [14] measuring mental health literacy in adolescents aged 14–16 [14]. The instrument consists of 50 items aiming to measure knowledge and attitudes to mental health across seven domains: (1) knowledge about mental health (12 items), (2) knowledge about mental health-promoting behaviors (6 items; previously known as “good mental health behaviors”), (3) stigma (6 items), (4) (lack of) self-stigma (6 items), (5) (lack of) avoidant coping (5 items), (6) help-seeking behaviors (7 items) and (7) social desirability (8 items). Participants are asked to rate their agreement with statements on a five-point Likert scale (Strongly Agree, Agree, Don’t Know, Disagree, Strongly Disagree) [14]. A correct answer for “Strongly Agree” or “Strongly Disagree” (as defined by the authors for factually correct answers or exhibiting minimal stigma) always received a score of 4. The response of “Don’t Know” always received a rating of 2. If responses deviated by one category (e.g., from “Agree” to “Strongly Agree”), a rating of 3 was given. Deviating by three categories (e.g., from “Disagree” to “Strongly Agree”) resulted in a rating of 1, by four categories in a rating of 0. The items were scored in a manner that higher scores represented positive attitudes or behaviors. Several items were reverse-scored and average scores were calculated for each subscale and adjusted for missing items. A total MHL score is calculated by summing each of the average subscales (excluding subscale social desirability).

### Phase 1: Translation and cultural adaptation of KAMHS-NL

The translation of the KAMHS was done following the guidelines by Guillemin and Beaton [18]. It consists of six steps: forward translation by two independent translators, synthesis, back translation, expert committee review, pre-testing and the formulation of the definitive translated version. The KAMHS was translated from the English to the Dutch language by two independent translators, of whom both are Dutch native speakers, and one has some expertise in mental health while the other does not. After synthesis of the two preliminary Dutch versions through consensus of the research team, a backward translation was performed. The backward translation was done by two translators whose native language is Dutch and who are proficient in the English language. Both are naïve in the construct of mental health. The differences between the original and translated version were discussed until resolved through consensus by the research team resulting in the Dutch translation of the KAMHS, the KAMHS-NL.

#### Sample

During the phase of pre-testing, content validity was established by conducting interviews (*n* = 16) with adolescents aged 13–15. Participants were students at two secondary schools in the Netherlands, with eight identifying as female (50%), seven as male (43%), and one as non-binary (7%). Three participants had parents of non-Dutch descent (19%).

#### Procedure

During the interviews, a ‘think aloud’ strategy was applied: The participants were first asked to fill in a questionnaire on their demographic information. They were then asked to complete the KAMHS-NL while articulating their thoughts aloud. The participants were then asked to comment on the relevance of each item, the comprehensiveness of the scale, and the comprehensibility of the scale instructions, items, and response options [17, 19]. The interviews were led by the first author (JMT) and a student assistant and was guided by a probing guide. The interviews were conducted at the school on-site and took on average 20 min. Each interview was audio recorded and field notes were made throughout and after each interview.

#### Data analysis

The interview data and field notes were processed and analyzed using Atlas.ti (Version 9). The transcripts of the interviews were read by the first author (JMT) and patterns concerning difficulties with items or instructions were noted. Thematic content analysis was performed by coding the verbatim transcripts of the interviews and then organizing the codes into thematic categories. Data were coded by JMT. Afterwards, the results were discussed in the research team and decisions were made on whether changes in the questionnaire were necessary. Based on the discussions, the scale was revised leading to a final version.

### Phase 2: Psychometric properties of KAMHS-NL

After the translation and cross-cultural adaptation process described in phase 1, a cross-sectional study was performed to assess the psychometric properties of the KAMHS. Reliability, construct and structural validity were assessed.

#### Sample

The study sample included *n* = 406 adolescents between ages 11 and 16 attending two different secondary schools in the Netherlands. The sample size meets the quality criteria stated by Terwee et al. (2007) which suggest a sample size of at least *n* = 100 for assessing construct and structural validity [20]. Participants were able to participate when they were between the ages 11 and 16.

#### Procedure and measures

A survey was administered to the participants. Study data were collected and managed using REDCap electronic data capture tools hosted at the University Medical Center Groningen [21, 22]. REDCap (Research Electronic Data Capture) is a secure, web-based software platform designed to support data capture for research studies. Data were collected in September and October 2022. Participants were asked to answer questions regarding their background, and to fill in the KAMHS-NL as well as several reference scales to evaluate construct validity. Reference scales that had no formally translated Dutch-language version (i.e., AMHSS and HSI) were translated to the Dutch language and adapted for the adolescent population by the authors of this study. Reference scales are described below.

##### Strengths and Difficulties Questionnaire (SDQ)

The SDQ is a brief behavioral screening questionnaire measuring emotional and behavioral problems, hereafter referred to as ‘mental health’, for children and adolescents (aged 4–18) containing 25 items divided into 5 scales of 5 items each: (1) emotional symptoms, (2) conduct problems, (3) hyperactivity/inattention, (4) peer relationship problems, and (5) prosocial behavior. Items are rated on a 3-point rating scale (0 = *not true*, 1 = *somewhat true*, and 2 = *certainly true*). Scores of the first four subscales are added together to generate a total difficulties score (0 to 40). Higher scores represent a higher degree of difficulties. The Dutch norm for a normal level of mental health problems in adolescents is a total score of 12 and lower [23]. The SDQ is a widely used and well-validated questionnaire in the scientific community [24].

##### Brief Resilience Scale (BRS)

The BRS is a six-item questionnaire measuring resilience that has been validated in different contexts. The items include statements such as *“I tend to bounce back quickly after hard times”* and “*I have a hard time making it through stressful events*”. The respondents are asked to indicate how well each statement describes their behavior and actions on a 5-point Likert-type scale, ranging from “1” = *does not describe me at all* to “5” = *describes me very well*. Item scores were calculated into an average total score. The BRS has shown consistently good psychometric properties [25–27].

##### WHO Wellbeing Index (WHO-5)

The World Health Organization-Five Well-Being Index (WHO-5) is a short self-reported measure of current mental wellbeing available in more than 30 languages. It is suitable for children aged 9 and above and has been found to have good construct validity. The WHO-5 consists of five statements, in which respondents rate on a 5-point Likert scale how often they experienced various feelings in the previous two weeks, ranging from “5” = *all of the time* to “0” = *at no time*. The total raw score, ranging from 0 to 25, is multiplied by 4 to give the final score, with 0 representing the worst imaginable well-being and 100 representing the best imaginable well-being [28].

##### Adolescent Mental Health Support Scale (AMHSS)

The AMHSS is a validated, brief self-report measure of adolescent mental health service use. It addresses two aspects of mental health service use: (1) desire for use of mental health services and (2) actual use of mental health services. Respondents are asked to indicate if during the past 12 months, they had the desire to talk to a school counselor, school therapist or school psychologist about emotional challenges or problems (answer options *Yes’*,* ‘No’*,* ‘Not sure’*). Then they are asked to indicate how often they have made use of mental health services during the past 12 months, ranging from psychologists, teachers, other trusted adults to parents or peers (answer options *zero times (0)*,* one time (1)*,* two or three times (2)*,* or four or more times (3)* [29]. For the purposes of this study, a sum score was computed representing the frequency of having made use of mental health services ranging from 0 to 30. Analyses with the AMHSS variable were performed including only participants with mental health problems as measured by the SDQ (score 13 or higher).

##### Help-Seeking Intentions (HSI) Vignette

A HSI vignette was used to assess help-seeking intentions, as in previous MHL studies. This regarded a vignette formulated by Jorm et al. (2000) describing a person (adolescent) who met ICD-10 and DSM-IV criteria for major depression with alcohol misuse [30]. The respondents were presented with 34 help sources (e.g., going to the GP, using vitamins and minerals, using antidepressants) that they had to rate as helpful (= 2), harmful (= 0), or neither (= 1). A correct identification of helpfulness or harmfulness of help-sources received a score of 2. If a helpful help-source was falsely indicated as harmful or a harmful one as helpful, a score of 0 was received. If a help-source was rated as neither harmful nor helpful, it yielded a score of 1. Scores for all items were calculated into an average total score, with higher scores indicating a greater sense of helpful or harmful help-sources.

##### Proximity (to someone with mental health problems)

Proximity (to someone with mental health problems) has been measured with the question “Do you know someone with mental health problems?” with the answer options “Yes”, “No”, or “I don’t know”. If answered with “yes”, the participants had the option to specify the person they know with a mental health problem and the kind of mental health problems. For this study, we dichotomized this variable into 0 = ‘No’/’I don’t know’ and 1 = ‘Yes’.

As background characteristics, we assessed age, gender (female, male, non-binary or other), descent (adolescent and parents’ country of birth: Dutch, Turkish, Moroccan, Surinamese, or other descent), school grade, and school level (lower secondary, intermediate secondary, higher secondary).

#### Data analysis

First, we assessed the background characteristics of the study sample. Second, construct validity was assessed by computing convergent and divergent Spearman correlations between subscales of KAMHS-NL and the reference scales. Convergent validity is demonstrated when the measure significantly correlates moderately to strongly (in the hypothesized direction) with other measures that should theoretically be related to one another. Divergent validity is shown when the measure has weak or no correlations with measures that should not be theoretically related [31]. Prior to the analysis, hypotheses regarding convergent and divergent correlations were determined based on the construct of MHL, its subdomains and formerly established correlations of related constructs [32], see Table 1 below. To account for the six instances of multiple testing (per subscale) the significance level of 0.05 was adjusted to 0.01.

Correlation coefficients between 0.10 and 0.30 were considered weak, between 0.30 and 0.50 as moderate, and higher than 0.50 as strong [33].

**Table 1:** Expected convergent and divergent Spearman correlations between KAMHS-NL subscales and related questionnaires

	SDQ	BRS	WHO-5	AMHSS	HSI	Proximity
Total KAHMS-NL Score	-	0	+	+	+	0
Mental health knowledge	-	0	+	0	+	+
Knowledge mental health-promoting behaviors	-	0	+	+	+	0
(Lack of) stigma	-	0	+	+	0	+
(Lack of) self-stigma	-	0	+	+	+	0
(Lack of) avoidant coping	-	0	+	+	+	0
Help-seeking behaviors	-	0	+	+	+	0
Social desirability	0	0	0	0	0	0

+ = expected positive correlation; - = expected negative correlation (convergent validity); 0 = no association expected (divergent validity)

KAMHS-NL = Knowledge and Attitudes to Mental Health Scales (Dutch); SDQ = Strengths and Difficulties Scale; BRS = Brief Resilience Scale; AMHSS = Adolescent Mental Health Support Scale; HSI = Help-Seeking Intentions Vignette

Third, we assessed the structural validity of the KAMHS-NL by performing factor analyses. A five-factor structure of the KAMHS was previously found by Simkiss et al. (2021) through a principal component analysis (PCA) and confirmed by a confirmatory factor analysis (CFA) [14]. We attempted to confirm this previously found structure in the Dutch sample. Confirmatory factor analysis was conducted on 28 items (excluding mental health knowledge scale items due to items being multifaceted) assuming correlations between MHL factors [34]. For the CFA, a robust maximum likelihood (MLR) estimator was employed for estimating parameters, alongside STDYX-standardization. Items 8 and 21 were not included in the model as they performed poorly in a preliminary CFA with 30 items, similar to the prior analysis in Simkiss, et al. (2021). The KAMHS subscales ‘Mental health knowledge’ and ‘Social desirability’ were excluded from factor analyses as the items of the former are multifaceted and the latter is not a structural component of MHL. Item loadings larger or equal to 0.40 were considered satisfactory [35]. In addition, internal consistency coefficients (McDonald’s omega) as well as correlation coefficients between factors were calculated.

The fit of the model (goodness-of-fit) was assessed using the Comparative Fit Index (CFI), Tucker Lewis Index (TLI) and the Root Mean Square Error of Approximation Value (RMSEA). CFI and TLI values above or equal to 0.90 and RMSEA values below or equal to 0.05 indicate a good model-fit [36]. If the model fit indices pointed towards inadequate fit for the five-factor model, we proceeded to assess the factor structure through an exploratory structural equation modeling (ESEM) of five factors, applying Target rotation. An ESEM is a statistical technique that combines elements of both exploratory factor analysis (EFA) and confirmatory factor analysis (CFA) allowing for cross-loadings [37]. MLR was also used in the ESEM, and the standardized parameter estimates (STDYX) were reported [38]. The goodness-of-fit indices of the two models were subsequently compared again considering values of CFI, TLI, and RSMEA. Chi-Square Test of Model Fit and RMSEA 90% Confidence Intervals were reported. Items that did not perform adequately (loadings smaller than 0.30) in the ESEM were removed in a subsequent analysis repeating the same CFA and ESEM strategy.

Construct validity analyses were performed using IBM Statistics 28. Factor analyses were performed using MPlus version 8.9.

#### Missing values

Prior to the analysis, participants with ten or more missing values on the KAMHS-NL items were excluded from analysis (23.2%) resulting in the presented sample size of *n* = 406. Missing values of the KAMHS in the final dataset were imputed under the assumption of missing at random through multiple imputation in SAS version 9.4 with the procedure Proc MI where the fully conditional specification (FCS) was used with the parametric method predictive mean matching (PMM), which assumes the existence of a joint distribution for all variables [39, 40]. The number of imputations was 50.

## Results

Results are presented separately per phase, i.e., of translation and validation.

### Phase 1: Translation and cultural adaptation of the KAMHS

After the translation procedure, the expert committee found that the Dutch version of the KAMHS was clear and comprehensible to the Dutch adolescent population.

The cognitive interviews with adolescents (n = 16) showed that the overall scale, instructions, response options, layout and almost all items were considered to be relevant, comprehensive, and comprehensible. The participants indicated that they mainly had difficulties with mental disorder-specific terms such as “generalized anxiety disorder” (item 13), “manic” (item 31), and “schizophrenia” (items 18 and 32). Item 38 (“Mental disorders are caused by people being wicked or bad”) caused some confusion among the adolescents as it was unclear if the mental disorder would be caused if the people themselves were being wicked or bad or if someone else behaved wickedly or bad towards them. Double negatives as seen in item 23 (‘If I had a mental disorder, I would not avoid socializing”) also caused some difficulties for the participants. After discussion with the research team and the authors of the original KAMHS, we made the following changes to the scale: 1) “Generalized anxiety disorder” was shortened to “anxiety disorder” (item 13) and 2) item 38 was slightly adjusted to “Mental disorders are caused *when* people behave wickedly or bad”. Finally, a final Dutch version of the KAMHS-NL was produced.

### Phase 2: Psychometric properties KAMHS-NL

#### Description of the sample

The sample for this study is described in Table 2 (*N* = 406). About half of the sample identified as female (54.2%). Most participants were of Dutch descent (58.9%), and 21.4% attended the highest secondary school level (VWO in Dutch), 42.4% attended intermediate secondary education (HAVO) and 35.7% attended the lower vocational education level (VMBO).

**Table 2:** Descriptive statistics of the sample

Variables	Total *N =* 406
Age *M* (in years*)*	12.7
**Gender** *n* (%) Female Male Non-binary or other	406 (100%) 220 (54.2%) 176 (43.3%) 10 (2.4%)
**Descent** *n* (%) Dutch Morocco/Turkey Surinam/Dutch Antilles Other OECD country Other non-OECD country	386 (100%) 239 (61.9%) 64 (16.6%) 16 (4.1%) 19 (4.9%) 48 (12.4%)
**School grade** *n* (%) First Second Third Fourth	403 (100%) 149 (37.0%) 142 (35.2%) 90 (22.3%) 22 (5.5%)
**School level** *n* (%) Lower secondary Intermediate secondary Higher secondary	404 (100%) 145 (35.9%) 172 (42.6%) 187 (21.5%)
**SDQ ≥ 13**	185 (45.6%)

Descriptive statistics for the used questionnaires and subscales in this study are presented in Table 3.

**Table 3:** Descriptive statistics for used questionnaires and subscales

	*N*	Items	Possible score range	Observed score range	Mean (SD)
**KAMHS-NL (total)**	**406**	50	0–24	7.38–20.65	14.10 (2.15)
Mental health knowledge	406	12	0–4	1.33–3.42	2.20 (0.28)
Knowledge mental health-promoting behaviors	406	6	0–4	0.67-4.00	2.57 (0.55)
(Lack of) stigma	406	6	0–4	1.17-4.00	2.53 (0.59)
(Lack of) self-stigma	406	6	0–4	0.00–4.00	2.14 (0.72)
(Lack of) avoidant coping	406	5	0–4	0.20-4.00	2.30 (0.66)
Help-seeking behaviors	406	7	0–4	0.00–4.00	2.33 (0.70)
Social desirability	406	8	0–4	0.25–3.75	1.95 (0.60)
**SDQ**	374	25	1–3	1.00–3.00	1.84 (0.91)
**BRS**	342	6	0–6	1.00–5.00	2.90 (0.74)
**WHO-5 Index**	350	5	0-100	0-100	45.55 (21.69)
**AMHSS**	141	10	0–30	0–29	6.06 (5.21)
**HSI Vignette**	306	34	0–2	0.15–1.91	1.45 (0.30)
**Proximity**	398	1	0–1 (no-yes)	0–1	0.28 (28% yes)

Note: KAMHS = Knowledge and Attitudes to Mental Health Scales; SDQ = Strengths and Difficulties Questionnaire; BRS = Brief Resilience Scale; HSI = Help-Seeking Intentions Vignette; AMHSS = Adolescent Mental Health Support Scale

#### Construct validity

Table 4 shows the convergent and divergent correlations of the KAMHS-NL subscales with reference scales. Seventeen of the 28 hypotheses regarding convergent correlations between the KAMHS-NL and the various reference scales were confirmed. The KAMHS-NL subscales ‘knowledge about mental health’ and ‘(lack of) self-stigma’ correlated weakly in the opposite direction as hypothesized with the SDQ and the WHO-5, not confirming our hypotheses. Also, expected convergent correlations between the KAMHS-NL and the AMHSS as well as the HSI (except for KAMHS-NL total score and ‘knowledge about mental health-promoting behaviors’ subscale) could not be confirmed. Convergent correlations between the KAMHS-NL and reference scales generally indicated significant associations, varying in strength from weak (.15) to strong (.50).

Eleven of the 20 hypotheses regarding divergent correlations were confirmed. Subscales of the KAMHS had unexpected significant correlations with the BRS and the KAMHS subscale ‘social desirability’ had unexpected associations with the SDQ, WHO-5, and proximity.

Divergent correlations between these KAMHS subscales and reference scales showed weak-to-moderate effect sizes (between 0.02 and 0.40).

A complete overview of the convergent and divergent correlation coefficients is presented in the Supplementary Materials.

**Table 4:** Expected and observed convergent and divergent Spearman correlations between KAMHS subscales and related questionnaires

	SDQ		BRS		WHO-5		AMHSS		HSI		Proximity	
	Ex	Ob	Ex	Ob	Ex	Ob	Ex	Ob	Ex	Ob	Ex	Ob
Total score	**-**	-	0	+	+	+	+	0	+	+	0	0
Mental health knowledge	-	0	0	0	+	-	0	0	+	0	+	+
Knowledge mental health-promoting behaviors	**-**	-	0	+	+	+	+	0	+	+	0	0
(Lack of) stigma	**-**	+	0	-	+	-	+	0	0	0	+	+
(Lack of) self-stigma	**-**	**-**	0	+	+	+	+	0	+	0	0	0
(Lack of) avoidant coping	**-**	-	0	0	+	+	+	0	+	0	0	0
Help-seeking behaviors	-	-	0	+	+	+	+	0	+	0	0	0
Social desirability	0	-	0	+	0	+	0	0	0	0	0	-
Number of confirmed hypotheses	5/8		2/8		5/8		2/8		4/8		7/8	

Note: Ex = Expected; Ob = Observed; + = positive correlation; - = negative correlation; 0 = no correlation

#### Structural validity

##### Confirmatory factor analysis (CFA)

The CFA showed that for twenty-four of the twenty-eight variables, items loaded with the corresponding factors above the recommended 0.40 cut-off which indicates good associations. Factor loadings were comparable to the original Welsh data for most items, except for 23 and 42. Items 37 and 45, which loaded poorly in the Welsh dataset, performed adequately in our analysis. The model had an insufficient goodness-of-fit (CFI = 0.86, TLI = 0.84, RMSEA = 0.053), so an exploratory structural equation modeling (ESEM) was performed. A complete overview of the factor loadings of the CFA28 model with five factors as well as internal consistency coefficients and factor correlations is presented in the Supplementary Materials.

##### Exploratory structural equation modeling (ESEM)

An ESEM was performed to find a better factor structure of the KAMHS in the Dutch sample for 5 factors (with 28 items; ESEM28). This resulted in a better goodness-of-fit as shown in Table 5. The CFI and TLI increased by more than 0.01 and RMSEA decreased by more than 0.015 in the ESEM28 compared to the CFA28 model indicating a better fitting ESEM28 model. The ESEM28 model showed good associations of the items and their designated factor. Some items also showed significant cross-loadings indicating associations with other factors (e.g., item 10 and Factor 1 ‘Help-Seeking Behaviors’). A complete overview of the factor loadings of the ESEM model with five factors is presented in the Supplementary Materials.

In the Supplementary Materials, we also present a re-estimation of the CFA and ESEM with 25 items, excluding items 38, 44, and 46 (CFA25 and ESEM25) as they did not perform adequately in the analysis with 28 items (item loadings smaller than 0.30). Table 5 shows the goodness-of-fit statistics of all models. As the goodness-of-fit statistics between ESEM28 and ESEM25 are comparable, we retain the model with 28 items for conceptual reasons, as these items may still provide content-level value to the KAMHS.

**Table 5:** Goodness-of-fit statistics for the models CFA28, ESEM28, CFA25 and ESEM25

	CFA28	ESEM28	CFA25	ESEM25
RMSEA (SD)	0.053 (0.001)	0.033 (0.002)	0.058 (0.001)	0.034 (0.002)
RMSEA 90% CI	0.048–0.059	0.033–0.034	0.058–0.058	0.034–0.035
CFI	0.86	0.96	0.85	0.96
TLI	0.84	0.93	0.83	0.94
Chi-Square Test of Model Fit (df)	725.62 (340)	359.79 (248)	629.15 (265)	272.41 (185)

CFA: Confirmatory factor analysis for 5-factor structure

ESEM: Exploratory Structural Equation Modeling

RMSEA = Root Mean Square Error of Approximation

CFI = Comparative Fit Index

TLI = Tucker-Lewis Index

SD = Standard Deviation

df = degrees of freedom

CI = Confidence Interval

## Discussion

The aim of the present study was to translate and culturally adapt a comprehensive instrument measuring adolescents’ mental health literacy to Dutch and assess its psychometric properties. Our findings show that the KAMHS-NL has good content validity, construct validity, and acceptable to good reliability. Furthermore, we generally confirm the structural validity of the KAMHS in the Dutch sample with its 5-factor structure.

We found the Dutch translation of the KAMHS to be relevant, comprehensive, and comprehensible, with only minor adaptations needed compared to the Welsh version. The minor adaptations that were made regarded specific formulations that were unclear to the target group due to cross-cultural translations, a common occurrence in studies of this nature. Establishing content validity through cognitive interviews has shown to be a valuable approach.

The construct validity of the KAMHS-NL was confirmed fulfilling most but not all its criteria. In line with previous research and our hypotheses, adolescents with higher levels of MHL showed better mental health. Thus, adolescents with better knowledge of mental health-promoting behaviors and help-seeking behaviors and lower levels of stigma and avoidant coping and may have better mental health [41–43]. However, our hypotheses were not supported in the MHL domains of mental health knowledge and self-stigma, revealing an inverse relationship. This suggests that adolescents with lower levels of mental health knowledge and higher levels of self-stigma tend to have better mental health. Regarding knowledge about mental health, this finding may be due to the way mental health knowledge is measured in the KAMHS assessing multi-faceted knowledge of causes, risk factors and symptoms of mental illness rather than the sole recognition of a specific mental illness as it is often done in other MHL studies [7]. Adolescents with poorer mental health may therefore be more knowledgeable of causes, risk factors, and symptoms of mental health knowledge, possibly by recognizing them in themselves. This finding highlights the importance of streamlining the definition of MHL and its related subdomains, for example by finding consensus for conceptualizing mental health knowledge. Researchers have yet to agree on how mental health knowledge should be measured, with either multi-dimensional constructs or a single construct. While mental health knowledge is deemed to be multi-faceted, it may be reasonable to classify the constructs into subscales and achieve the reliability for each individual subscale.

Our finding that adolescents who hold more self-stigmatizing attitudes have better mental health goes against our hypotheses but is reflected in the rather contradicting literature on the mechanisms of (self-)stigma. Some previous studies suggested that experiencing mental health problems leads to less internalized stigmatizing attitudes while others have shown the opposite [44–46]. Our study shows that adolescents with better mental health may be aware of and agree with stereotypes towards individuals with mental health problems or illness and indicate that they would think less of themselves if they were ever to experience such problems. Our findings also confirmed our hypothesis that individuals who had greater direct or indirect experience with mental health problems or illness had better mental health knowledge and lower levels of stigma [46]. This implies that reducing the distance to individuals with mental health problems and illness leads to better mental health knowledge and fewer stigmatizing attitudes of youth.

Not in line with previous research and our hypotheses, our findings showed no association between MHL and use of mental health services among participants with mental health problems and help-seeking intentions. An explanation for this finding may be that overall use of mental health services is low [3, 47], largely reducing the power to detect associations. Consequently, the absence of significant correlations between MHL and mental health service utilization in our study may stem from our sample of adolescents not accessing mental health services, irrespective of their levels of MHL. Additionally, our expected convergent correlations between KAMHS subdomains and help-seeking intentions could also not be fully supported despite the often-hypothesized associations between the concept of help-seeking intentions with stigma, knowledge of mental health-promoting behaviors, and help-seeking behaviors [48–50]. However, the reference scale for help-seeking intentions in this study had a limited scoring range which may have reduced the power to identify the associations between these constructs. Also in contrast to our hypotheses, we did find significant, but weak associations between dimensions of MHL and the concept of resilience. This is also in line with research that has been published after we formulated our hypotheses [51]. No other previous research has shown associations between MHL and resilience. Taken together, these findings indicate a relationship between the concepts of MHL and resilience. This implies that improved MHL among adolescents may lead to more effective coping with stress which is important for school and future work outcomes [52].

Furthermore, we unexpectedly found associations between the KAMHS subscale social desirability and mental health, resilience, and proximity. These results imply that adolescents who tend to report more socially desirable responses also tend to report better mental health, higher resilience and knowing someone with mental health problems. This may have led to a slight overestimation of the self-reported information on mental health and resilience, and even to socially desirable responses on other KAMHS subscales, potentially affecting their construct validity. Only few studies have thus far investigated the relationship between social desirability and mental health [53, 54] and further research investigating the role of social desirability in mental health (literacy) questionnaires may contribute to improved insights and better assessment in this regard.

We found the KAMHS-NL to generally have acceptable to good internal consistency. The only exception to this were the subscales ‘knowledge about mental health’ and ‘(lack of) avoidant coping’. These findings are in line with the previous study of Simkiss et al. (2021) that also found poor internal consistencies for these subscales. For the knowledge about mental health subscale, this may be due to the nature of the items that measure aspects of knowledge regarding mental health and disorders rather than attitudes. For the avoidant coping subscale, the small number of items may contribute to the poor internal consistency. Also, some of this subscale’s items relate to one’s knowledge of coping strategies which offers another explanation of these results. It’s important to note that these findings have not affected the subsequent analyses in this study, given that the mental health knowledge subscale and two avoidant coping items were intentionally excluded from factor analyses.

Finally, we could generally confirm the structural validity of the KAMHS-NL. Our findings show that the instrument’s items measure the intended underlying construct of MHL and its subdomains in the better fitting ESEM model as the majority of the items load satisfactory on the intended factors. The ESEM model revealed some non-loading items, and we observed items belonging to the subscale ‘(lack of) self-stigma’ loading on the subscale ‘help-seeking behaviors’ instead or vice versa. This confirms the positive relationship between these two domains, as help-seeking behaviors are often influenced by self-stigma [14]. However, it is important to emphasize, as previously recommended by Simkiss, et al. (2021), that despite this relationship, help-seeking behaviors and self-stigma are distinct concepts with unique contributions to other domains of MHL and should be treated as such. Nevertheless, our findings suggest the need to closely examine poorly loading or clustering items and potentially reformulate or omit items.In addition, some items (e.g., items 23 and 38) might be worded too difficult for the adolescent population in either language which is why they consistently perform poorly [14]. This was also confirmed by the participants of the cognitive interviews who had some difficulties with understanding and responding to these items. A post-hoc analysis utilizing Item Response Theory was performed that confirmed our findings regarding the items that perform suboptimal. Future research is encouraged to re-examine the performance of items (especially for items 8, 21, 10, 38, 44 and 46) through qualitative and Item Response Theory analyses and confirm their factor loadings in larger samples. If future research confirms our results, it may be considered to reallocate items to another subscale or completely remove items from the KAMHS.

### Strengths and limitations

Our study had several important strengths, the first being its sample that was highly representative for Dutch adolescents regarding gender identity, descent, grades, and educational levels. As a result, our study’s findings hold significant potential for high generalizability among Dutch adolescents. Second, our study has achieved the validation of the KAMHS in a new cultural setting, successfully including adolescents between 11 and 16 years old, an age group that has been underrepresented in previous research [14]. Last, we explored the structure of the KAMHS-NL using a more robust technique of factor analysis allowing for cross-loadings between factors (ESEM). We believe that this has led to a more adequate estimation of the model fit.

Our study also has a number of limitations. This study’s cross-sectional design restricted the possibilities to evaluate response consistency within the same sample over time (test-retest reliability). As a result, we were unable to assess the stability and repeatability of participants’ responses. To overcome this limitation in future research, incorporating a longitudinal approach would be beneficial, allowing for the examination of response stability and changes over an extended period. Also, specific reference scales used in this study had a restricted scoring range, which may have impacted the ability to identify associations between constructs. This might have led to underestimation of some associations.

### Implications for practice and research

This study indicates that the KAMHS-NL is a valid instrument to assess mental health literacy in Dutch adolescents, comprehensively assessing all domains of MHL from a positive mental health perspective. The KAMHS-NL is therefore promising for use in research and also in practice, e.g., in schools and mental health care organizations, to identify youth with low levels of MHL. The instrument may also be used to highlight domains of MHL that require additional attention in mental health education efforts.

Future research is encouraged to further enhance the evidence base of the KAMHS and substantiate its psychometric properties by assessing different groups and larger samples, thereby establishing measurement invariance. Positive findings may then invite translation and evaluation of the KAMHS in various other languages and cultural contexts. This would further contribute to streamlining MHL measurement and evidence across different contexts.

## Conclusion

This study demonstrates the KAMHS’ ability to measure levels of MHL validly and reliably in Dutch adolescents. As a result, it holds promising potential for detecting MHL levels in adolescents. In future studies, it is recommended to validate the scale in other settings to facilitate high-quality research and enable meaningful comparisons across different contexts.

### Electronic supplementary material

Below is the link to the electronic supplementary material.

Supplementary Material 1

Supplementary Material 2

Supplementary Material 3

Supplementary Material 4

Supplementary Material 5

## Data Availability

The datasets used and/or analysed during the current study are available from the corresponding author on reasonable request.

## Abbreviations

MHL: Mental health literacy
KAMHS: Knowledge and Attitudes to Mental Health Scales
COSMIN: COsensus-based Standards for the selection of health Measurement INstruments
SDQ: Strengths and Difficulties Questionnaire
BRS: Brief Resilience Scale
WHO-5: WHO Index 5
HIS: Help-Seeking Intentions
AMHSS: Adolescent Mental Health Support Scale
CFI: Comparative Fit Index
TLI: Tucker Lewis Index
RMSEA: Root Mean Square Error of Approximation Value
PCA: Principal component analysis
CFA: Confirmatory factor analysis
MLR: Robust Maximum Likelihood
ESEM: Exploratory structural equation modeling
CI: Confidence Interval
FCS: Fully Conditional Specification
PMM: Predictive Mean Matching
IRT: Item Response Theory
