# Supplementary material for: Psychometric properties of the Knowledge and Attitudes to Mental Health Scales in a Dutch sample (KAMHS-NL): A comprehensive mental health literacy measure in adolescents
Source: BMC Public Health. 2024 Jul 25;24:1995. doi: 10.1186/s12889-024-19371-3 (PMC11282802; doi:10.1186/s12889-024-19371-3)
Supplement: Supplementary file 2 — Supplementary Material 2 [file 12889_2024_19371_MOESM2_ESM.docx]

**Supplementary** **Table 2.** Standardized loadings (β), Standard Error (S.E.), and omega internal consistency (ω) for CFA model: Five factors on 28 items of the KAMHS-NL (CFA28).

| Item no. | Factor and item in full | | | | | ω | Standardized loadings (S.E.) | |
| --- | --- | --- | --- | --- | --- | --- | --- | --- |
| Factor 1: Help-Seeking Behaviors | | | | | | 0.76 |  | |
| 5 | I wouldn’t tell anyone if I had a mental health problem in case they made fun of me | | | | |  | 0.59 (0.04) | |
| 11 | For me, it would be easy to ask for help for a mental health problem | | | | |  | 0.55 (0.05) | |
| 24 | If I had a mental health problem, I would try to hide it from everyone | | | | |  | 0.69 (0.03) | |
| 42 | If I had a mental health problem, I would be happy to tell my teacher or school counsellor | | | | |  | 0.38 (0.06) | |
| 46 | If I had a mental health problem, I would not tell friends and family | | | | |  | 0.51 (0.04) | |
| 1 | I am confident that I could ask for help if I had a mental health problem | | | | |  | 0.61 (0.04) | |
| 45 | It’s best not to tell anyone about your mental health problems | | | | |  | 0.52 (0.04) | |
| Factor 2: (Lack of) Self-Stigma | | | | | | 0.83 |  | |
| 10 | If I had a mental disorder, I would not feel ashamed | | | | |  | 0.40 (0.05) | |
| 23 | If I had a mental disorder, I would not avoid socialising | | | | |  | 0.29 (0.06) | |
| 25 | I would feel a failure if I had a mental disorder | | | | |  | 0.76 (0.03) | |
| 33 | If I had a mental disorder, I would feel worthless like I had failed my family | | | | |  | 0.85 (0.02) | |
| 43 | I would feel weak if I had a mental disorder | | | | |  | 0.76 (0.02) | |
| 49 | If I had a mental disorder, I would feel I’d let everyone down | | | | |  | 0.84 (0.02) | |
| Factor 3: (Lack of) Stigma | | | | | | 0.73 |  | |
| 2 | If my friend had a mental disorder, I would avoid them | | | | |  | 0.51 (0.05) | |
| 16 | I would not like to be in the same classroom as someone with a mental disorder | | | | |  | 0.70 (0.04) | |
| 27 | I wouldn’t want to marry or date a person with a mental disorder | | | | |  | 0.57 (0.04) | |
| 36 | I would be happy for a person with a mental disorder to come to my house | | | | |  | 0.64 (0.04) | |
| 17 | I would feel comfortable sitting next to a person with a mental disorder | | | | |  | 0.60 (0.04) | |
| 38 | Mental disorders are caused by people being wicked or bad | | | | |  | 0.29 (0.05) | |
| Factor 4: Knowledge Mental Health-Promoting Behaviors | | | | | | 0.65 |  | |
| 34 | The same things that help our physical health also help our mental health | | | | |  | 0.40 (0.05) | |
| 35 | Sometimes things that stress you should be faced head-on | | | | |  | 0.47 (0.05) | |
| 39 | A good night’s sleep is good for your mental health | | | | |  | 0.63 (0.04) | |
| 44 | Regular exercise has no effect on your mental health | | | | |  | 0.25 (0.06) | |
| 50 | Talking about your feelings can help with mental health problems | | | | |  | 0.56 (0.05) | |
| 37 | Healthy eating helps you maintain good mental health | | | | |  | 0.58 (0.05) | |
| Factor 5: (Lack of) Avoidant Coping | | | | | | 0.62 |  | |
| 6 | It’s often best to ignore problems and hope they go away | | | | |  | 0.73 (0.05) | |
| 47 | I do my best not to think about my problems | | | | |  | 0.51 (0.05) | |
| 40 | The best way to cope with problems is not to think about them | | | | |  | 0.52 (0.06) | |
| Factor correlations | | | | | | | | |
| 1 | | **1** | **2** | **3** | **4** | | | 5 |
| 2 | | **0.73** | 1 |  |  | | |  |
| 3 | | 0.03 | 0.07 | 1 |  | | |  |
| 4 | | **0.61** | **0.41** | 0.11 | 1 | | |  |
| 5 | | **0.55** | **0.51** | 0.08 | 0.29 | | | 1 |
