# Supplementary material for: Psychometric properties of the Knowledge and Attitudes to Mental Health Scales in a Dutch sample (KAMHS-NL): A comprehensive mental health literacy measure in adolescents
Source: BMC Public Health. 2024 Jul 25;24:1995. doi: 10.1186/s12889-024-19371-3 (PMC11282802; doi:10.1186/s12889-024-19371-3)
Supplement: Supplementary file 4 — Supplementary Material 4 [file 12889_2024_19371_MOESM4_ESM.docx]

**Supplementary Table 1**. Convergent and divergent Spearman correlations between KAMHS subscales and related questionnaires (^1^Kendall’s tau-b for correlations with binary variables), 99% confidence intervals are presented in brackets.

|  | SDQ  (N = 374) | | | BRS  (N = 342) | | | WHO-5  (N = 350) | | | AMHSS  (N = 141)^2^ | | | HIS  (N = 306) | | | Proximity^1^  (N = 398) | |
| --- | --- | --- | --- | --- | --- | --- | --- | --- | --- | --- | --- | --- | --- | --- | --- | --- | --- |
|  | Ex | Ob | Ex | | Ob | Ex | | Ob | Ex | | Ob | Ex | | Ob | Ex | | Ob |
| KAMHS total score | **-** | -0.45*  [-0.56, -0.34] | 0 | | 0.29*  [0.15, 0.42] | + | | 0.38*  [0.26, 0.50] | + | | 0.00  [-0.22, 0.23] | + | | 0.21*  [0.06, 0.35] | 0 | | -0.02  [-0.10, 0.07] |
| Mental Health Knowledge | - | 0.11  [-0.03, 0.24] | 0 | | -0.05  [-0.19, 0.09] | + | | -0.15*  [-0.29, -0.01] | 0 | | 0.05  [-0.18, 0.27] | + | | 0.11  [-0.05, 0.25] | + | | 0.16*  [0.07, 0.24] |
| Knowledge Mental Health-Promoting Behaviors | **-** | - 0.28*  [-0**.**40, -0.15] | 0 | | 0.16*  [0.02, 0.29] | + | | 0.36*  [0.23, 0.48] | + | | 0.05  [-0.18, 0.27] | + | | 0.27*  [0.12, 0.41] | 0 | | -0.05  [-0.14, 0.03] |
| (Lack of) Stigma | **-** | 0.16*  [0.02, 0.29] | 0 | | -0.19*  [-0.33, -0.05] | + | | -0.16*  [-0.29, -0.02] | + | | 0.10  [-0.13, 0.31] | 0 | | 0.07  [-0.08, 0.22] | + | | 0.25*  [0.17, 0.33] |
| (Lack of) Self-Stigma | **-** | -0.41*  [0.52, -0.29] | 0 | | 0.40*  [0.27, 0.51] | + | | 0.37*  [0.27, 0.46] | + | | -0.08  [-0.29, 0.15] | + | | 0.12  [-0.04, 0.26] | 0 | | -0.07  [-0.16, -0.01] |
| (Lack of) Avoidant Coping | **-** | -0.41*  [-0.52, -0.29] | 0 | | 0.14  [-0.01, 0.27] | + | | 0.22*  [0.11, 0.32] | + | | -0.06  [-0.27, 0.17] | + | | 0.09  [-0.06, 0.26] | 0 | | -0.08  [-0.17, 0.00] |
| Help-Seeking Behaviors | - | -0.49*  [-0.59, -0.38] | 0 | | 0.35*  [0.22, 0.47] | + | | 0.50*  [0.39, 0.60] | + | | 0.13  [-0.10, 0.34] | + | | 0.14  [-0.01, 0.29] | 0 | | -0.08  [-0.16, -0.01] |
| Social Desirability | 0 | -0.35*  [-0.46, -0.22] | 0 | | 0.20*  [0.06, 0.33] | 0 | | 0.26*  [0.13, 0.39] | 0 | | -0.12  [-0.33, 0.11] | 0 | | -0.04  [-0.19, 0.11] | 0 | | -0.13*  [-0.21, -0.04] |
| Number of confirmed hypotheses | 5/8 | | | 2/8 | | | 5/8 | | | 2/8 | | | 4/8 | | | 7/8 | |

Note: Ex = Expected; Ob = Observed. + = positive correlation; - = negative correlation; 0 = no correlation. * = significant at the 0.01 level (2-tailed). ^1^ Kendall’s tau-b correlation coefficient. ^2^ included when SDQ ≥ 13.
