# Supplementary material for: Psychometric properties of the Knowledge and Attitudes to Mental Health Scales in a Dutch sample (KAMHS-NL): A comprehensive mental health literacy measure in adolescents
Source: BMC Public Health. 2024 Jul 25;24:1995. doi: 10.1186/s12889-024-19371-3 (PMC11282802; doi:10.1186/s12889-024-19371-3)
Supplement: Supplementary file 5 — Supplementary Material 5 [file 12889_2024_19371_MOESM5_ESM.docx]

**Supplementary Table 5.** Standardized loadings (β) and standard errors (S.E.) for a 5-factor Exploratory Structural Equation Modelling model: Five factors on 25 items of the KAMHS.

| **Item no.** | **Item in full** | **Factor 1** | **Factor 2** | **Factor 3** | **Factor 4** | **Factor 5** |
| --- | --- | --- | --- | --- | --- | --- |
| **5** | I wouldn’t tell anyone if I had a mental health problem in case they made fun of me | **0.45 (0.08)** | **0.23**  **(0.07)** | 0.03  (0.05) | **-0.10 (0.06)** | **0.16 (0.06)** |
| **11** | For me, it would be easy to ask for help for a mental health problem | **0.63 (0.08)** | 0.04 (0.06) | **-0.12 (0.05)** | 0.13 (0.06) | -0.06 (0.06) |
| **24** | If I had a mental health problem, I would try to hide it from everyone | **0.61 (0.10)** | **0.18 (0.08)** | 0.02 (0.05) | -0.04 (0.06) | 0.11 (0.06) |
| **42** | If I had a mental health problem, I would be happy to tell my teacher or school counsellor | **0.33**  **(0.09)** | -0.03  (0.08) | 0.09  (0.07) | **0.18**  **(0.08)** | -0.02  (0.08) |
| **45** | It’s best not to tell anyone about your mental health problems | 0.12 (0.11) | 0.12 (0.08) | **0.11 (0.05)** | **0.24 (0.07)** | **0.37 (0.07)** |
| **1** | I am confident that I could ask for help if I had a mental health problem | **0.38**  **(0.08)** | 0.07  (0.06) | **-0.15**  **(0.05)** | **0.30**  **(0.06)** | 0.11  (0.06) |
| **10** | If I had a mental disorder, I would not feel ashamed | **0.49 (0.08)** | 0.14 (0.08) | **0.18 (0.06)** | -0.00 (0.07) | -0.08 (0.08) |
| **23** | If I had a mental disorder, I would not avoid socialising | **0.32 (0.09)** | 0.07 (0.09) | 0.09 (0.06) | 0.10 (0.07) | -0.01 (0.08) |
| **25** | I would feel a failure if I had a mental disorder | **0.18 (0.06)** | **0.70 (0.06)** | -0.04 (0.03) | **-0.09 (0.04)** | -0.02 (0.05) |
| **33** | If I had a mental disorder, I would feel worthless like I had failed my family | -0.04 (0.05) | **0.86 (0.05)** | -0.01 (0.03) | **0.08 (0.04)** | -0.02 (0.04) |
| **43** | I would feel weak if I had a mental disorder | -0.07 (0.06) | **0.84 (0.05)** | 0.05 (0.04) | -0.02 (0.05) | -0.05  (0.05) |
| **49** | If I had a mental disorder, I would feel I’d let everyone down | -0.05 (0.06) | **0.82 (0.05)** | 0.00 (0.03) | 0.07 (0.05) | 0.07 (0.04) |
| **2** | If my friend had a mental disorder, I would avoid them | **-0.19 (0.07)** | 0.05 (0.06) | **0.50 (0.05)** | 0.07  (0.06) | 0.09 (0.07) |
| **16** | I would not like to be in the same classroom as someone with a mental disorder | 0.05 (0.07) | 0.01 (0.06) | **0.68 (0.05)** | -0.03 (0.05) | 0.04 (0.05) |
| **27** | I wouldn’t want to marry or date a person with a mental disorder | -0.09 (0.07) | 0.06 (0.05) | **0.59 (0.05)** | **-0.12 (0.06)** | 0.02 (0.05) |
| **36** | I would be happy for a person with a mental disorder to come to my house | **0.12 (0.06)** | -0.07 (0.05) | **0.65 (0.05)** | 0.08 (0.05) | -0.07 (0.06) |
| **17** | I would feel comfortable sitting next to a person with a mental disorder | -0.05 (0.08) | 0.02 (0.07) | **0.62 (0.05)** | 0.00 (0.06) | -0.10 (0.06) |
| **34** | The same things that help our physical health also help our mental health | 0.13  (0.08) | 0.01  (0.07) | 0.03  (0.06) | **0.38**  **(0.08)** | **-0.15**  **(0.07)** |
| **35** | Sometimes things that stress you should be faced head-on | -0.02 (0.09) | 0.06 (0.07) | 0.00 (0.06) | **0.52 (0.08)** | -0.06 (0.07) |
| **37** | Healthy eating helps you maintain good mental health | **0.16 (0.07)** | 0.04 (0.07) | -0.03 (0.05) | **0.52 (0.07)** | **-0.14 (0.06)** |
| **39** | A good night’s sleep is good for your mental health | 0.03 (0.08) | 0.08 (0.06) | -0.04 (0.04) | **0.57 (0.08)** | -0.05 (0.07) |
| **50** | Talking about your feelings can help with mental health problems | -0.01 (0.07) | -0.04 (0.05) | 0.08 (0.04) | **0.56 (0.06)** | **0.29 (0.06)** |
| **6** | It’s often best to ignore problems and hope they go away | 0.04 (0.06) | **0.15 (0.06)** | 0.00 (0.04) | 0.07 (0.05) | **0.53 (0.06)** |
| **47** | I do my best not to think about my problems | **0.24 (0.07)** | 0.07 (0.08) | **-0.16 (0.05)** | **-0.18 (0.06)** | **0.50 (0.09)** |
| **40** | The best way to cope with problems is not to think about them | 0.01 (0.07) | -0.10 (0.06) | **0.11 (0.04)** | 0.06 (0.06) | **0.67 (0.08)** |
| **Factor correlations** | | | | | | |
| 1 |  | 1 | **0.51** | 0.00 | **0.32** | 0.11 |
| 2 |  |  | 1 | 0.06 | 0.27 | **0.37** |
| 3 |  |  |  | 1 | 0.12 | 0.11 |
| 4 |  |  |  |  | 1 | 0.20 |
| 5 |  |  |  |  |  | 1 |

Values with corresponding significant loadings (or correlations) (p ≤ 0.05) are written in bold
